# Altered Placental Ezrin Expression in Gestational Diabetes Mellitus: An Immunohistochemical and Bioinformatic Analysis

**DOI:** 10.3390/diagnostics16152404

**Published:** 2026-07-30

**Authors:** Fırat Aşır, Ebru Gökalp Özkorkmaz, Nilüfer Dönmezdil, Senem Çetin Duran, Fırat Şahin, Tuğcan Korak, Elif Ağaçayak

**Affiliations:** 1 Department of Histology and Embryology, Faculty of Medicine, Dicle University, 21280 Diyarbakır, Turkey; 2 Faculty of Health Sciences, Ankara Yıldırım Beyazıt University, Çubuk, 06760 Ankara, Turkey; 3 Department of Histology and Embryology, Faculty of Medicine, Van Yüzüncü Yıl University, 65080 Van, Turkey; 4 Division of Infertility, Turan Çetin In-Vitro Fertilization Center, Çukurova, 01360 Adana, Turkey; 5 Department of Medical Biology, Faculty of Medicine, Kocaeli University, 41380 İzmit, Turkey; 6 Department of Gynecology and Obstetrics, Faculty of Medicine, Dicle University, 21280 Diyarbakır, Turkey

**Keywords:** gestational diabetes mellitus, placenta, ezrin, radixin, moesin, bioinformatics

## Abstract

**Background/Objectives**: Gestational diabetes mellitus (GDM) is associated with placental structural and functional alterations that may impair trophoblast integrity and maternal–fetal exchange. Ezrin, radixin, and moesin (ERM) proteins are membrane–cytoskeleton linker proteins involved in microvillous organization, cell polarity, and signal transduction. This study aimed to investigate placental ERM protein expression in GDM and to explore ERM-associated molecular pathways through bioinformatic analysis. **Methods**: This prospective observational study included placental tissues obtained from 40 women with GDM and 40 healthy pregnant women. Immunohistochemical staining for ezrin, radixin, and moesin was performed on paraffin-embedded placental sections, and quantitative image analysis was conducted using QuPath software (version 0.7.0) to calculate H-scores. Correlation and receiver operating characteristic (ROC) analyses were performed to evaluate associations between ERM expression and clinical parameters. In addition, bioinformatic analyses were conducted using the publicly available GSE154414 placental transcriptomic dataset to investigate ERM-associated signaling pathways and interaction networks. **Results**: Ezrin immunoreactivity was significantly increased in the GDM group compared with controls (118.63 ± 20.56 vs. 107.95 ± 17.41, *p* = 0.026), whereas Radixin and Moesin expression levels did not differ significantly between groups (*p* > 0.05). Ezrin expression demonstrated significant positive correlations with maternal body mass index, birth weight, fasting glucose, and HbA1c levels. ROC analysis demonstrated modest discriminatory performance of the Ezrin H-score for distinguishing GDM from control pregnancies (AUC = 0.645, *p* = 0.018). Bioinformatic analyses identified enrichment of ERM-associated pathways involved in cytoskeletal remodeling, inflammatory signaling, and protein phosphorylation-related cellular processes. **Conclusions**: Placental Ezrin expression was significantly increased in women with GDM and was positively associated with maternal glycemic parameters. These findings suggest that altered Ezrin expression may reflect adaptive cytoskeletal remodeling in diabetic placentas. ERM-associated signaling pathways may contribute to placental responses to metabolic stress in GDM.

## 1. Introduction

Gestational diabetes mellitus (GDM) is one of the most common metabolic disorders of pregnancy and is associated with adverse maternal and fetal outcomes, including fetal macrosomia, neonatal metabolic disturbances, preeclampsia, and long-term metabolic disease risk [1,2]. Many of these complications are closely linked to structural and functional alterations in the placenta, which plays a central role in maternal–fetal nutrient and gas exchange [3,4]. Previous studies have demonstrated that GDM placentas exhibit villous immaturity, syncytial knot formation, stromal edema, vascular congestion, fibrinoid deposition, and trophoblastic dysfunction [1,3,5].

The syncytiotrophoblast layer contains a highly specialized microvillous membrane that depends on cytoskeletal integrity to maintain trophoblast structure and membrane organization [6,7]. Among the proteins involved in this process, the ezrin/radixin/moesin (ERM) family functions as a membrane–cytoskeleton linker complex regulating cell polarity, adhesion, membrane stability, and signal transduction [8,9]. ERM proteins undergo phosphorylation-dependent activation and interact with both membrane-associated proteins and filamentous actin [10,11].

Ezrin is a major structural component of epithelial microvilli and has been associated with trophoblast migration, invasion, and membrane organization [9,12]. Radixin and moesin similarly contribute to cytoskeletal stabilization and membrane dynamics [13,14]. Because placental tissues in GDM are exposed to chronic hyperglycemia [15], oxidative stress, and inflammatory activation [16,17], alterations in ERM protein expression may occur as part of trophoblastic adaptation and cytoskeletal remodeling. However, data regarding ERM protein expression in diabetic placentas remain limited.

Therefore, the present study aimed to investigate the immunohistochemical expression patterns of ezrin, radixin, and moesin in placentas obtained from women with GDM and to evaluate their potential association with GDM-related placental alterations using quantitative image analysis and bioinformatic approaches. In addition, network-based bioinformatic analysis was performed to identify shared molecular targets and signaling pathways associated with ERM-centered interaction networks in GDM.

## 2. Materials and Methods

### 2.1. Study Design and Patient Selection

The study protocol was approved by the Non-Interventional Clinical Research Ethics Committee of Dicle University Faculty of Medicine (approval date: 18 June 2025; approval number: 2025/262), and written informed consent was obtained from all participants. This prospective case–control observational study was conducted between August 2025 and January 2026 at the Department of Obstetrics and Gynecology, Dicle University Faculty of Medicine. Placental tissues were obtained from 80 pregnant women, including 40 healthy controls and 40 patients diagnosed with GDM. Only singleton term pregnancies resulting in live births between 37 + 0 and 41 + 6 weeks of gestation were included. Women aged 18–40 years were eligible for enrollment.

GDM was diagnosed during the current pregnancy using a one-step 75 g oral glucose tolerance test according to the International Association of Diabetes and Pregnancy Study Groups criteria [18]. GDM was defined by at least one of the following plasma glucose values: fasting ≥ 92 mg/dL, 1 h ≥ 180 mg/dL, or 2 h ≥ 153 mg/dL. The control group consisted of healthy pregnant women without systemic disease or obstetric complications.

**Figure 1:**
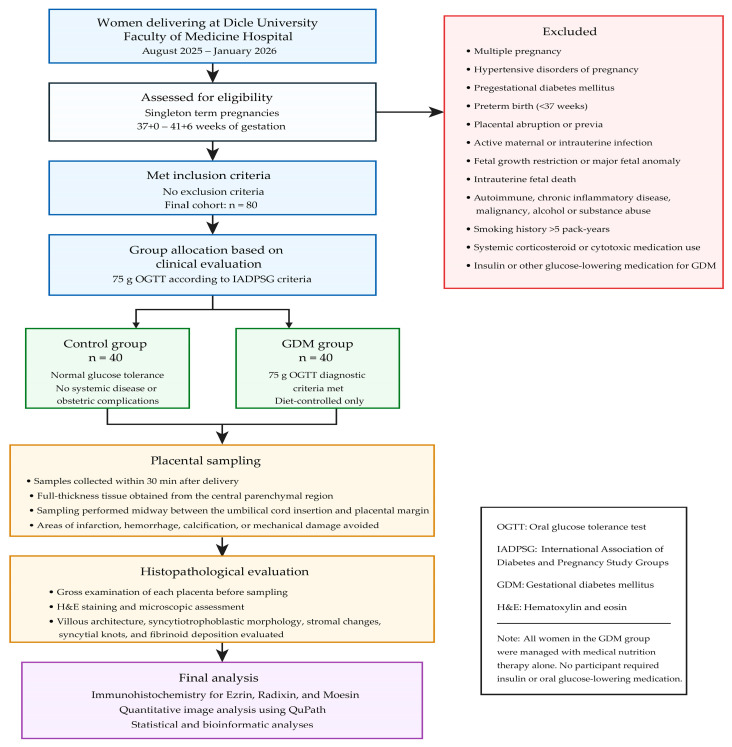
Flowchart of patient selection, placental sampling, and study workflow.

Exclusion criteria included pre-GDM, multiple pregnancy, hypertensive disorders of pregnancy, placental pathology, fetal growth restriction, active maternal infection, major fetal anomaly, intrauterine fetal death, autoimmune or chronic inflammatory disease, malignancy, smoking history >5 pack-years, alcohol or substance abuse, systemic corticosteroid use, cytotoxic medication use, insulin therapy or other glucose-lowering medications for GDM, and maternal body mass index >40 kg/m^2^. Placental tissue samples were collected within 30 min after delivery. To reduce treatment-related heterogeneity, only women with diet-controlled GDM managed with medical nutrition therapy were included. Patients requiring insulin therapy or other glucose-lowering medications during pregnancy were excluded, as pharmacological treatment may independently influence placental structure and molecular expression profiles. To minimize sampling bias, all placentas were sampled according to the same standardized protocol by obtaining full-thickness tissue from the central placental parenchyma, midway between the umbilical cord insertion and the placental margin, while avoiding grossly abnormal areas. All control placentas were obtained from uncomplicated singleton term pregnancies. Following gross examination and histopathological evaluation, no unexpected pathological findings requiring exclusion were identified. The overall study design, patient selection process, placental sampling strategy, and analytical workflow are summarized in Figure 1.

### 2.2. Placental Tissue Sampling and Processing

Placental tissue samples were collected within 30 min after delivery. After removal of the fetal membranes and umbilical cord, the placenta was examined macroscopically. Tissue samples were obtained from the central parenchymal region, avoiding areas of gross infarction, hemorrhage, calcification, or mechanical damage. Full-thickness placental tissue sections including the maternal and fetal surfaces were sampled from representative cotyledons. The samples were fixed in 10% neutral buffered formalin for 24 h and then processed routinely through graded alcohols and xylene before being embedded in paraffin blocks. For routine histopathological evaluation, paraffin-embedded placental tissue blocks were sectioned at 4–5 μm thickness and mounted on glass slides. Sections were deparaffinized in xylene, rehydrated through graded ethanol solutions, and stained with hematoxylin and eosin (H&E) according to standard histological procedures. The stained sections were examined under a Zeiss Imager A2 light microscope (Carl Zeiss, Oberkochen, Germany), and representative images were captured at 20× magnification. Histopathological evaluation focused on overall villous architecture, syncytiotrophoblastic morphology, villous stromal changes, syncytial knot formation, and fibrinoid deposition.

### 2.3. Immunohistochemical Analysis

Paraffin-embedded placental sections (4–5 μm) were mounted on poly-L-lysine-coated slides, deparaffinized in xylene, and rehydrated through descending alcohol series. Endogenous peroxidase activity was blocked using hydrogen peroxide solution (TA-015-HP, Thermo Fisher Scientific, Fremont, CA, USA), followed by incubation with Ultra V Block solution (TA-015-UB, Thermo Fisher Scientific, Fremont, CA, USA) to prevent nonspecific binding. Sections were incubated overnight at 4 °C with primary antibodies against ezrin (sc-58758), radixin (sc-6408), and moesin (sc-13122) (Santa Cruz Biotechnology, Dallas, TX, USA). After incubation with a biotinylated secondary antibody (TP-015-BN, Thermo Fisher Scientific) and streptavidin–peroxidase solution (TS-015-HR, Thermo Fisher Scientific), immunoreactivity was visualized using diaminobenzidine chromogen (TA-001-HCX, Thermo Fisher Scientific). Sections were counterstained with Harris hematoxylin and examined using a Zeiss Imager A2 light microscope (Zeiss Inc., Jena, Germany) [19].

### 2.4. Quantitative Evaluation of Immunohistochemical Staining

Digital image analysis was performed using QuPath software (version 0.7.0). Representative images were captured under identical microscope settings, and at least five non-overlapping high-power fields were analyzed for each case. Positive-staining areas for ezrin, radixin, and moesin were quantified using color deconvolution and threshold-based detection algorithms. H-scores were calculated according to the following formula: H-score = ∑Pi(i + 1), where Pi represents the percentage of positively stained cells and i indicates staining intensity (0–3). The H-score method was selected because it combines staining intensity and the proportion of immunopositive cells into a single semi-quantitative measure, providing a more comprehensive assessment of protein expression than either parameter alone. In addition, digital image analysis using QuPath improves objectivity and reproducibility by minimizing observer-dependent variability. Final values were obtained by averaging all analyzed fields. All analyses were performed independently by two blinded histologists, and mean values were used for statistical analysis [20].

### 2.5. Bioinformatic and Pathway Enrichment Analysis

Publicly available placental transcriptomic data associated with GDM were obtained from the Gene Expression Omnibus (GEO) database (GSE154414). The dataset included four GDM placental samples and four healthy control placental samples. Differential gene expression analysis was performed using the processed gene expression matrix following platform-provided normalization and log_2_ transformation. Differentially expressed genes were initially identified using a nominal *p* value < 0.05. To account for multiple comparisons, false discovery rate (FDR) correction was applied using the Benjamini–Hochberg procedure, and adjusted *p* values were used to assess statistical significance. Because only a limited number of genes remained significant after FDR correction, exploratory visualization and downstream biological interpretation were primarily based on nominally significant genes, whereas FDR-adjusted results were considered when interpreting statistical robustness. Analysis focused on ezrin/radixin/moesin (ERM)-associated and cytoskeleton-related genes, including EZR, MSN, RDX, ICAM1, VCAM1, ROCK1, RHOA, ACTB, MMP2, MMP9, TNF, and IL6. Volcano plot analysis was performed to visualize differential gene expression profiles between groups [21].

### 2.6. GDM-Associated ERM Interaction Network and Pathway Analysis

Protein–protein interaction network analysis was performed using the STRING database to investigate signaling pathways associated with ERM proteins in GDM [22]. GDM-associated proteins together with ezrin, radixin, and moesin were used to construct interaction networks with a medium confidence score and 300 additional interactors. Networks were imported into Cytoscape software (version 3.10.4), and overlapping proteins between the GDM network and each ERM-centered network were identified. Functional enrichment analyses, including Gene Ontology (GO), Kyoto Encyclopedia of Genes and Genomes (KEGG), and Reactome pathway analyses, were performed using the Enrichr platform. Pathways with *p* < 0.05 were considered statistically significant, and the top 10 enriched pathways were included in the analysis [23,24].

### 2.7. Statistical Analysis

All statistical analyses were performed using IBM SPSS Statistics version 25.0 (IBM Corp., Armonk, NY, USA). Data distribution was assessed using the Shapiro–Wilk test, histograms, Q–Q plots, and skewness/kurtosis values. Normally distributed continuous variables were expressed as mean ± standard deviation and compared using the independent samples *t*-test. Non-normally distributed variables were expressed as median [interquartile range] and compared using the Mann–Whitney U test. Categorical variables were presented as frequencies and percentages and compared using the chi-square test or Fisher’s exact test, as appropriate. Correlations between immunohistochemical H-scores and clinical parameters were evaluated using Spearman’s rank correlation analysis. Receiver operating characteristic (ROC) curve analysis was performed to assess the discriminatory performance of ERM protein expression, and the area under the curve (AUC), optimal cut-off values, sensitivity, and specificity were calculated. To determine whether the association between Ezrin expression and GDM was independent of potential confounding factors, analysis of covariance (ANCOVA) was performed with maternal age, maternal body mass index, and gestational age included as covariates. A two-sided *p* value < 0.05 was considered statistically significant.

## 3. Results

### 3.1. Clinical and Demographic Characteristics of the Study Groups

A total of 80 placental samples were included in the study, consisting of 40 control cases and 40 patients diagnosed with gestational GDM in Table 1. Maternal age was significantly higher in the GDM group compared to the control group (*p* < 0.001). Similarly, gravidity, parity, maternal body mass index, birth weight, fasting glucose levels, OGTT 1-h and 2-h glucose values, and HbA1c levels were significantly increased in the GDM group (all *p* < 0.05). Gestational age at delivery was significantly lower in the GDM group compared to controls (*p* < 0.001). Placental weight tended to be higher in the GDM group; however, this difference did not reach statistical significance (*p* = 0.053). The fetal-to-placental weight ratio was additionally calculated to assess placental efficiency. No significant difference was observed between the control and GDM groups (median [min–max]: 5.67 [3.98–7.33] vs. 5.72 [4.42–8.29], respectively; *p* = 0.294). In terms of neonatal sex distribution, male neonates were significantly more frequent in the GDM group compared to the control group (*p* = 0.014).

**Table 1:** Demographic and Clinical Characteristics of the Study Groups.

Variable	Control (*n* = 40)	GDM (*n* = 40)	*p*-Value
Maternal age (years)	29.30 ± 3.20	32.54 ± 3.70	<0.001
Gestational age (weeks)	39.21 ± 0.59	38.67 ± 0.75	<0.001
Gravidity	2.00 [1.00–3.00]	3.00 [2.00–4.00]	<0.001
Parity	1.00 [0.00–2.00]	2.00 [1.00–3.00]	<0.001
Maternal BMI (kg/m^2^)	27.50 [25.65–29.70]	29.60 [27.95–31.95]	0.003
Placental weight (g)	588.92 ± 68.42	624.17 ± 90.06	0.053
Birth weight (g)	3311.28 ± 256.74	3646.22 ± 236.17	<0.001
Fetal-to-placental weight ratio	5.67 [3.98–7.33]	5.72 [4.42–8.29]	0.294
Fasting glucose (mg/dL)	83.79 ± 4.83	101.67 ± 5.81	<0.001
OGTT 1h (mg/dL)	135.55 [123.05–143.57]	198.50 [180.52–207.93]	<0.001
OGTT 2h (mg/dL)	123.20 [111.65–128.07]	171.95 [156.78–180.07]	<0.001
HbA1c (%)	5.22 ± 0.17	5.90 ± 0.26	<0.001
Male/Female, n	13/27	25/15	0.014

Normally distributed variables are presented as mean ± standard deviation and were analyzed using Student’s *t*-test. Non-normally distributed variables are presented as median [interquartile range] and were analyzed using the Mann–Whitney U test. Categorical variables were analyzed using the chi-square test. Statistical significance was accepted as *p* < 0.05.

### 3.2. Histopathological Evaluation of Placental Tissue

**Figure 2:**
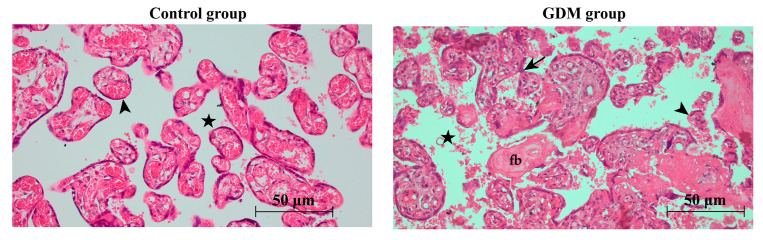
Representative H&E-stained placental sections from control and GDM pregnancies. Representative examples of the histopathological features observed in each group are shown. Images were acquired at ×20 magnification (scale bar = 50 μm). ★, intervillous space; arrowhead, syncytiotrophoblastic layer; arrow, syncytial knot; fb, fibrinoid deposition.

Histopathological examination of hematoxylin and eosin-stained placental sections demonstrated preserved villous architecture in the control group. Terminal villi exhibited regular morphology with a thin syncytiotrophoblastic layer, compact villous stroma, and evenly distributed fetal capillaries. Fibrinoid deposition and syncytial knots were minimal (Figure 2). In contrast, placentas from the GDM group exhibited focal distortion of villous architecture characterized by irregular villous contours, stromal loosening consistent with edema, increased syncytial knot formation, focal fibrinoid deposition, and irregular thickening of the syncytiotrophoblastic layer (Figure 2). These histopathological alterations were consistent with structural remodeling of placental villi associated with GDM.

### 3.3. Immunohistochemical Expression Patterns of ERM Proteins

**Figure 3:**
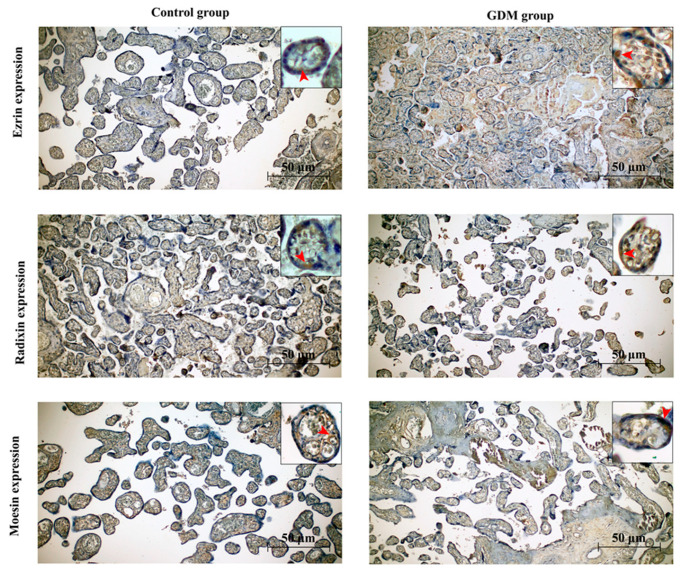
Representative immunohistochemical staining of Ezrin, Radixin, and Moesin in placental villi from control and GDM pregnancies. Ezrin, Radixin, and Moesin immunoreactivity was predominantly localized to the villous trophoblastic layer with membranous and cytoplasmic staining. Semi-quantitative H-score analysis demonstrated significantly increased Ezrin expression in GDM placentas compared with controls (*p* < 0.05), whereas Radixin and Moesin expression did not differ significantly between groups (*p* > 0.05). Red arrowheads indicate representative immunopositive staining. Scale bar = 50 μm, Magnification: 20X.

Ezrin immunoreactivity was predominantly localized in the villous trophoblastic layer, showing membranous and cytoplasmic staining patterns in both groups. Compared with controls, the GDM group demonstrated stronger and more diffuse Ezrin immunoreactivity, particularly along syncytiotrophoblastic surfaces. Semi-quantitative analysis confirmed significantly increased Ezrin expression in the GDM group (*p* < 0.05) (Figure 3). Radixin immunoreactivity was mainly localized along villous trophoblastic membranes and demonstrated similar staining patterns in both groups. Semi-quantitative analysis showed no significant difference in Radixin expression between the control and GDM groups (*p* > 0.05) (Figure 3). Moesin immunoreactivity was observed predominantly in villous trophoblastic regions with membranous and cytoplasmic staining patterns. Although staining distribution appeared relatively more diffuse in GDM placentas, Moesin expression did not differ significantly between groups (*p* > 0.05) (Figure 3).

### 3.4. Quantitative Analysis of Placental ERM Protein Expression

Immunohistochemical analysis demonstrated a significant increase in placental Ezrin expression in the GDM group compared to the control group (Table 2). The mean Ezrin H-score was significantly elevated in patients with GDM (118.63 ± 20.56) relative to controls (107.95 ± 17.41) (*p* = 0.026). Although Radixin H-score values were numerically higher in the GDM group, the difference between groups did not reach statistical significance (*p* = 0.184). Similarly, no statistically significant difference was observed in Moesin H-score values between the control and GDM groups (*p* = 0.769). These findings suggest that Ezrin may be more prominently associated with placental alterations occurring in GDM compared to other ERM family proteins.

### 3.5. Correlation Between ERM Protein Expression and Clinical Parameters

Correlation analysis demonstrated significant positive associations between placental Ezrin H-score and several clinical parameters associated with GDM (Table 3). Ezrin expression showed moderate positive correlations with maternal age, maternal BMI, birth weight, fasting glucose levels, and HbA1c values (all *p* < 0.05). The strongest correlations were observed between Ezrin expression and HbA1c levels (r = 0.498, *p* < 0.001) as well as fasting glucose levels (r = 0.471, *p* < 0.001), suggesting a close relationship between placental Ezrin expression and maternal glycemic status. In contrast, Radixin and Moesin H-score values did not demonstrate significant correlations with most clinical parameters evaluated.

### 3.6. ROC Curve Analysis

**Figure 4:**
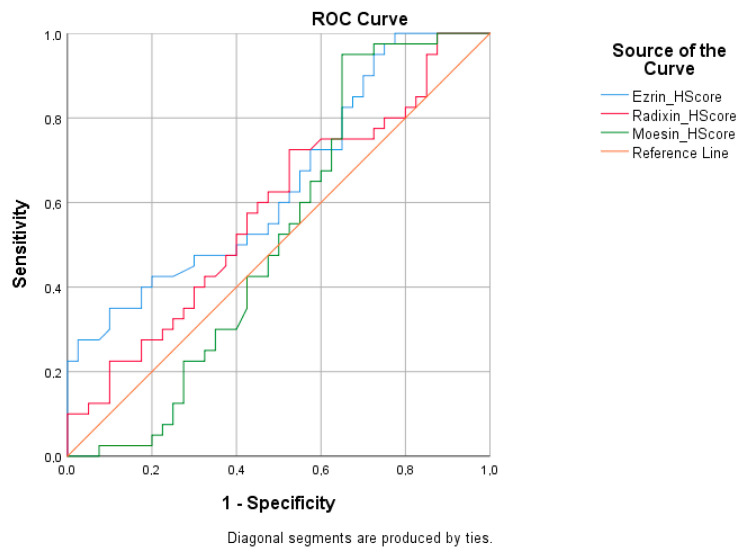
ROC curves showing the discriminatory performance of placental Ezrin, Radixin, and Moesin H-scores for differentiating GDM from control pregnancies. Curves are shown for each ERM protein, with the diagonal reference line indicating the expected performance of random classification (AUC = 0.5). Diagnostic performance was evaluated using the AUC, with corresponding sensitivity and specificity values reported in the Results section.

ROC curve analysis was performed to evaluate the discriminatory ability of placental ERM protein expression levels for distinguishing GDM cases from controls (Figure 4). Ezrin H-score demonstrated moderate but statistically significant discriminatory performance, with an AUC value of 0.645 (95% CI: 0.525–0.766, *p* = 0.018). The optimal cut-off value for Ezrin was >112.25, yielding 65.0% sensitivity and 45.0% specificity. In contrast, Radixin and Moesin H-scores did not show significant discriminatory performance. The AUC values were 0.582 (95% CI: 0.457–0.707, *p* = 0.200) for Radixin and 0.519 (95% CI: 0.392–0.647, *p* = 0.765) for Moesin. The optimal cut-off values were >116.60 for Radixin and >92.05 for Moesin.

To determine whether the observed difference in placental Ezrin expression was independent of potential confounding factors, an ANCOVA was performed with maternal age, maternal body mass index, and gestational age included as covariates. After adjustment, placental Ezrin H-score remained significantly higher in the GDM group than in the control group (adjusted mean: 123.93 vs. 111.75; F = 5.550, *p* = 0.021, partial η^2^ = 0.069). None of the covariates were significantly associated with Ezrin expression.

### 3.7. Transcriptomic Analysis of ERM-Associated Genes in GDM

**Figure 5:**
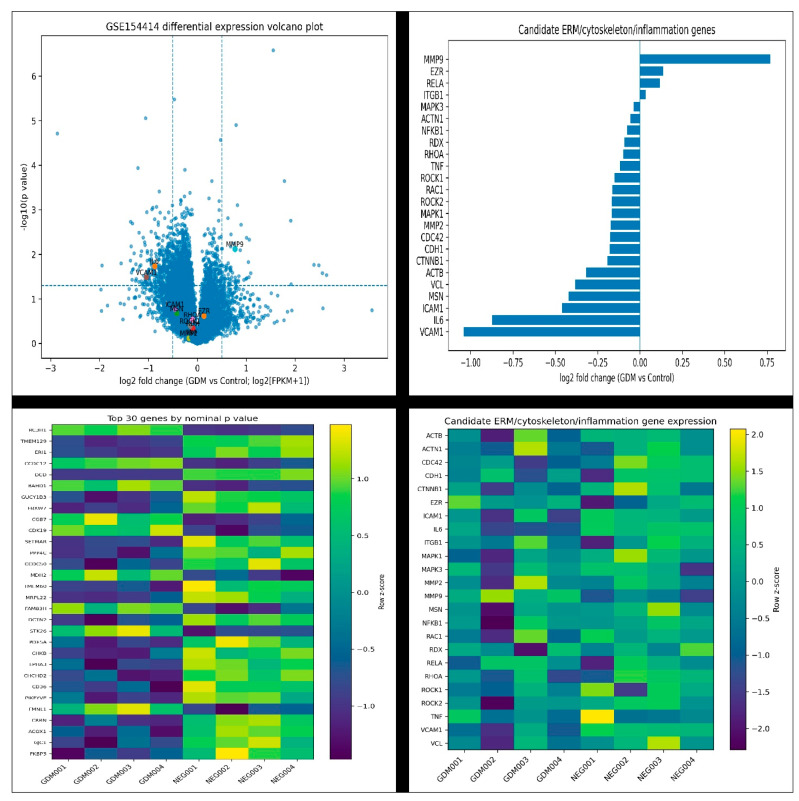
Transcriptomic analysis of placental tissues from GDM and control pregnancies using the GSE154414 dataset. (**A**) Volcano plot showing differentially expressed genes between GDM and control placentas. Upregulated genes are shown in red, downregulated genes in blue, and non-significant genes in gray. (**B**) Heatmap illustrating the relative expression patterns of selected ERM-associated and functionally related genes across individual placental samples. Color intensity represents relative gene expression after normalization.

Differential gene expression analysis of the GSE154414 dataset identified 410 nominally altered genes in GDM placentas, including 64 upregulated and 346 downregulated genes. However, only a limited number of genes remained significant after false discovery rate correction, indicating relatively modest transcriptomic alterations (Figure 5). Evaluation of ERM-associated genes demonstrated mild expression changes in EZR, MSN, and RDX transcripts. In contrast, several ERM-related inflammatory and cytoskeleton-associated genes, including MMP9, VCAM1, and IL6, showed altered expression profiles in GDM placentas (Figure 5). Volcano plot and heatmap analyses demonstrated partial clustering differences between GDM and control samples, particularly among genes associated with inflammatory signaling, cellular adhesion, and cytoskeletal organization (Figure 5).

### 3.8. Reactome Pathway Enrichment Analysis of GDM-Associated ERM Networks

**Figure 6:**
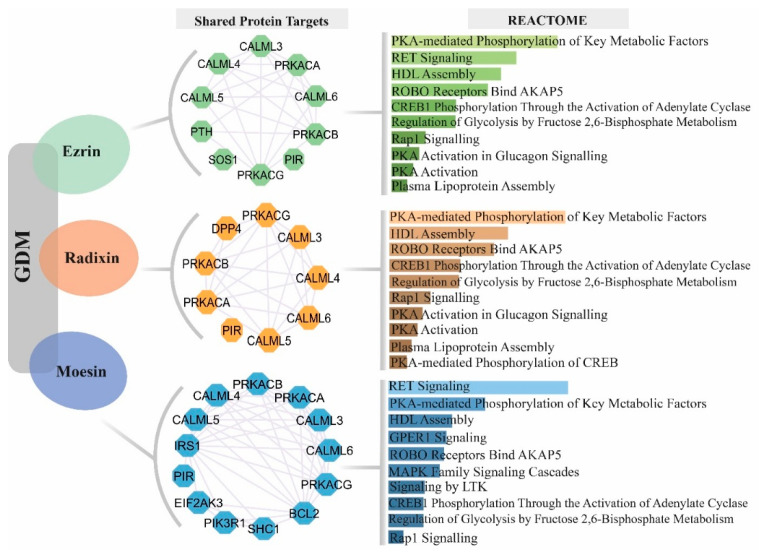
Protein–protein interaction and Reactome pathway enrichment analysis of ERM-associated networks in GDM. Left panels show the PPI networks generated for Ezrin, Radixin, and Moesin using STRING. Middle panels illustrate the overlapping protein targets shared between GDM-associated proteins and each ERM-centered interaction network. Right panels present the top 10 significantly enriched Reactome pathways (*p* < 0.05) identified from the overlapping protein sets. Together, these analyses highlight biological processes potentially linking ERM proteins with placental metabolic adaptation, cytoskeletal organization, and intracellular signaling in GDM.

The intersection analysis between GDM-associated proteins and EZR-centered interaction networks identified 10 common proteins, including PTH, PIR, SOS1, CALML family proteins, and PKA-associated catalytic subunits (PRKACA, PRKACB, and PRKACG). Reactome pathway enrichment analysis demonstrated significant enrichment in pathways related to PKA-mediated phosphorylation, RET signaling, HDL assembly, CREB1 phosphorylation, glycolytic regulation, Rap1 signaling, and ROBO–AKAP5 interactions (Figure 6). Similarly, the radixin-centered interaction network identified 9 shared proteins, including PIR, DPP4, CALML family proteins, and PKA-associated catalytic subunits. Enriched pathways were largely associated with PKA-mediated signaling, HDL and plasma lipoprotein assembly, CREB1 phosphorylation, glycolytic regulation, Rap1 signaling, and ROBO–AKAP5 interactions (Figure 6). The moesin-centered interaction network identified 13 common proteins, including PIR, EIF2AK3, PIK3R1, SHC1, BCL2, IRS1, CALML family proteins, and PKA-associated catalytic subunits. Reactome pathway analysis revealed enrichment in RET signaling, PKA-mediated phosphorylation, GPER1 signaling, MAPK family signaling cascades, LTK signaling, glycolytic regulation, CREB1 phosphorylation, and Rap1 signaling (Figure 6).

## 4. Discussion

The present study investigated the immunohistochemical expression of ezrin, radixin, and moesin proteins in placental tissues obtained from women with GDM. The principal finding was that placental Ezrin expression was significantly increased in the GDM group, whereas Radixin and Moesin expression levels did not differ significantly between groups. In addition, Ezrin expression demonstrated significant positive correlations with fasting glucose and HbA1c levels, suggesting a potential association between Ezrin expression and maternal metabolic status.

The placenta undergoes continuous structural and functional adaptation during pregnancy, and chronic exposure to hyperglycemia, oxidative stress, and low-grade inflammation in GDM may induce substantial trophoblastic and vascular alterations [25,26]. Previous studies have demonstrated that diabetic placentas exhibit villous remodeling, syncytial knot formation, stromal edema, vascular abnormalities, and altered membrane transport activity [27,28,29]. Because syncytiotrophoblast microvilli are highly dependent on membrane–cytoskeleton interactions, alterations in ERM protein expression may reflect adaptive cytoskeletal remodeling in response to diabetic stress [30,31,32].

Ezrin is a major membrane–cytoskeleton linker protein involved in cell polarity, membrane organization, and microvillus stability [33]. Experimental studies have demonstrated that Ezrin contributes to trophoblast migration, invasion, and cytoskeletal organization [34,35]. Tabrizi et al. [35] reported that phosphorylated Ezrin plays a critical role in extravillous trophoblast motility and invasion, supporting the biological relevance of ERM proteins in placental structural dynamics. In the present study, increased placental Ezrin immunoreactivity may therefore reflect an adaptive trophoblastic remodeling response that could contribute to the preservation of syncytiotrophoblast integrity under hyperglycemic conditions.

Maternal hyperglycemia is known to induce oxidative stress, inflammatory signaling, and metabolic reprogramming within the placenta, leading to the activation of intracellular pathways involved in cytoskeletal remodeling and cellular adaptation [36,37]. Among these, the cAMP/PKA/CREB1 signaling axis has been implicated in trophoblast glucose metabolism, differentiation, and stress-responsive gene regulation [38]. CREB1-mediated transcriptional activation may contribute to increased expression of cytoskeleton-associated proteins, including Ezrin, thereby promoting maintenance of trophoblast architecture under hyperglycemic conditions [39]. In parallel, Rap1 signaling regulates integrin activation, cell–cell adhesion, and actin cytoskeleton organization, processes in which Ezrin functions as a key membrane–cytoskeleton linker [40]. Consistent with these observations, our transcriptomic enrichment analyses identified significant involvement of both the cAMP/PKA/CREB1 and Rap1 pathways, supporting the hypothesis that increased Ezrin expression may represent an adaptive cytoskeletal response to the metabolic stress imposed by GDM rather than an isolated molecular alteration.

The observed correlations between Ezrin expression and fasting glucose, HbA1c, and birth weight are consistent with a relationship between placental Ezrin expression and maternal metabolic stress. Increased Ezrin expression in placentas from patients with higher glycemic parameters may reflect enhanced cytoskeletal activity and altered trophoblastic membrane dynamics in diabetic pregnancies. Mandò et al. [41] further demonstrated that GDM placentas exhibit altered mitochondrial bioenergetics and oxidative stress responses associated with maternal metabolic dysfunction, supporting the concept of adaptive placental remodeling under diabetic conditions. In contrast, Radixin and Moesin expression levels remained relatively stable, suggesting that Ezrin may play a more prominent role than Radixin or Moesin in placental adaptation to GDM-associated stress.

Bioinformatic analyses further supported the involvement of ERM-associated signaling pathways in GDM placentas. Enrichment analyses consistently identified pathways related to PKA/cAMP-mediated signaling, CREB1 phosphorylation, Rap1 signaling, glycolytic regulation, and cytoskeletal organization. These findings are biologically relevant because cAMP/PKA signaling has been implicated in placental glucose metabolism and trophoblast function. Chi et al. [42] demonstrated that cAMP/PKA/CREB1 signaling regulates glucose metabolism and GLUT1 expression in placental trophoblasts, linking this pathway to placental insulin resistance and metabolic dysfunction in GDM. Similarly, Yoshie et al. reported that Rap1 signaling modulates trophoblast invasion and growth factor-mediated cytoskeletal signaling through AKT, ERK, and p38MAPK pathways [43]. Collectively, these findings suggest that ERM-associated signaling networks may contribute to placental remodeling by linking metabolic stress with cytoskeletal adaptation and trophoblast dysfunction in GDM pregnancies [44].

The moderate discriminatory performance identified in the ROC analysis (AUC = 0.645) indicates that placental Ezrin expression alone is insufficient for clinical use as a diagnostic biomarker for GDM. Therefore, the ROC findings should be considered exploratory and interpreted with caution until validated in larger independent cohorts. Nevertheless, the observed associations with maternal glycemic parameters suggest that Ezrin expression may provide mechanistic insight into placental stress and cytoskeletal remodeling rather than immediate diagnostic utility. Importantly, the observed alterations in Ezrin expression should be interpreted alongside conventional histopathological findings and established clinical diagnostic criteria. Immunohistochemical evaluation is intended to provide mechanistic insight into placental remodeling rather than to serve as a standalone diagnostic approach for GDM.

This study has several limitations. First, the relatively modest sample size and the single-center case–control design may limit the generalizability of our findings. Second, the cross-sectional evaluation of placental tissue obtained at delivery precludes assessment of temporal changes in ERM protein expression throughout gestation and does not permit causal inference. Third, protein expression was evaluated exclusively by immunohistochemistry without complementary validation using quantitative techniques such as Western blotting or quantitative PCR. Moreover, only total Ezrin, Radixin, and Moesin expression was assessed; therefore, the phosphorylation-dependent activation status of ERM proteins could not be determined. Finally, no functional validation experiments, such as trophoblast cell culture models, gene silencing or overexpression approaches, or mechanistic pathway analyses, were performed. Consequently, the observed associations should be considered descriptive rather than causal. In addition, the correlation and ROC analyses should be interpreted cautiously because they were exploratory and performed in a relatively modest cohort without external validation. Future multicenter studies integrating molecular validation, phospho-protein analyses, and functional experiments are warranted to confirm the reproducibility of these findings and to clarify the mechanistic role and potential clinical relevance of Ezrin in placental remodeling associated with GDM.

Although Ezrin expression was significantly increased in GDM placentas, the diagnostic performance observed in the present study was modest, indicating that Ezrin alone is unlikely to serve as a clinically useful biomarker for placental maladaptation or GDM. Rather than functioning as a standalone diagnostic marker, increased Ezrin expression may reflect adaptive cytoskeletal remodeling in response to the metabolic stress associated with maternal hyperglycemia [45]. Accordingly, Ezrin may be better considered as one component of a broader molecular signature that incorporates established clinical parameters, histopathological findings, and additional placental biomarkers rather than as a standalone indicator. Future prospective studies integrating multiple molecular markers are warranted to determine whether composite biomarker panels can improve the identification and characterization of placental maladaptation in GDM.

## 5. Conclusions

Placental Ezrin expression was significantly increased in women with GDM and was positively associated with maternal glycemic parameters. In contrast, Radixin and Moesin expression levels did not differ significantly between groups. Bioinformatic analyses further supported the involvement of ERM-associated pathways related to PKA/cAMP signaling, Rap1 signaling, metabolic regulation, and cytoskeletal remodeling. Although Ezrin expression was significantly increased in GDM placentas, these findings should be interpreted as evidence of placental adaptation and cytoskeletal remodeling in conjunction with conventional histopathological assessment and clinical diagnosis, rather than as a diagnostic marker for GDM. Further molecular and functional studies are needed to clarify the precise role of ERM proteins in diabetic placental dysfunction.

## Figures and Tables

**Table 2:** Comparison of Placental ERM Protein Expression Between Groups.

Variable	Control (*n* = 40)	GDM (*n* = 40)	*p*-Value
Ezrin H-score	107.95 ± 17.41	118.63 ± 20.56	0.026
Radixin H-score	117.42 ± 17.06	122.11 ± 18.84	0.184
Moesin H-score	92.76 ± 14.48	93.88 ± 16.11	0.769

Data are presented as mean ± standard deviation. Group comparisons were performed using Student’s *t*-test or Mann–Whitney U test according to data distribution. Statistical significance was accepted as *p* < 0.05.

**Table 3:** Correlation Analysis Between ERM Protein Expression and Clinical Parameters.

Variable	Ezrin H-Score	Radixin H-Score	Moesin H-Score
Maternal age	r = 0.284, *p* = 0.011	r = 0.143, *p* = 0.207	r = 0.051, *p* = 0.655
Maternal BMI	r = 0.337, *p* = 0.002	r = 0.168, *p* = 0.137	r = 0.084, *p* = 0.459
Birth weight	r = 0.352, *p* = 0.001	r = 0.154, *p* = 0.173	r = 0.072, *p* = 0.528
Fasting glucose	r = 0.471, *p* < 0.001	r = 0.219, *p* = 0.051	r = 0.061, *p* = 0.592
HbA1c	r = 0.498, *p* < 0.001	r = 0.198, *p* = 0.079	r = 0.039, *p* = 0.731

Correlation analyses were performed using Spearman rank correlation analysis. Statistical significance was accepted as *p* < 0.05.

## Data Availability

The data presented in this study are available on request from the corresponding author due to patient privacy.
